# The Evaluation of Oral Health in Patients Undergoing Dental Treatment During the COVID-19 Pandemic

**DOI:** 10.3390/jcm13237216

**Published:** 2024-11-28

**Authors:** Bartosz Bielecki-Kowalski, Oliwia Kowalczyk, Maja Podziewska, Paulina Agier, Aleksandra Kroc-Szczepkowska, Marcin Kozakiewicz

**Affiliations:** 1 Department of Maxillofacial Surgery, Medical University of Lodz, 113 Żeromskiego Str., 90-549 Lodz, Poland; aleksandra.szczepkowska-kroc@office365.umed.pl (A.K.-S.); marcin.kozakiewicz@office365.umed.pl (M.K.); 2 Student Scientific Circle of Maxillofacial Surgery, Medical University of Lodz, 113 Żeromskiego Str., 90-549 Lodz, Poland; oliwia.kowalczyk@stud.umed.lodz.pl (O.K.); maja.podziewska@stud.umed.lodz.pl (M.P.); paulina.agier@stud.umed.lodz.pl (P.A.)

**Keywords:** COVID-19, vaccination, DMFT index, oral cavity health, dental treatment, place of residence

## Abstract

**Background**: The association between oral cavities and the SARS-CoV-2 virus is an issue commonly analyzed and studied. In our study, the relationship between the dental status and social environment of patients receiving treatment during the coronavirus pandemic and COVID-19 vaccination was analyzed. **Methods**: This retrospective study was based on 2034 dental records obtained from the Institute of Dentistry of the Medical University of Lodz. The collected data pertaining to caries enabled the assessment of the oral cavity health of individual patients and could be compared with the undergoing vaccination against COVID-19. **Results**: The statistically significant results showed that unvaccinated patients compared to vaccinated patients had more teeth with caries, less teeth extracted due to caries, more teeth in total, and lower DMFT and dental treatment indexes. It was shown that, with increased age, the value of the DMFT index increases. Statistically significant differences between patients living in rural and urban areas were as follows: rural residents showed lower DMFT and filling indexes. **Conclusions**: No direct relationship between the vaccination of patients and oral health has been proven. Statistically, the group of vaccinated patients are significantly older than the unvaccinated, and the significant differences between the above groups are most likely due to the difference in the average age of patients in the two groups. Our study showed a lower average DMFT index and a higher treatment intensity index in people from the countryside compared to those living in a large city. In this respect, our study stands in opposition to the existing research findings.

## 1. Introduction

Coronavirus 2, a virus from the Coronaviridae family, causes COVID-19, a disease that has become a pandemic since 2019 and caused a significant number of deaths worldwide [1]. Glycosylated spike proteins coat the surface of SARS-CoV-2, which binds to the host cell receptor-specific angiotensin-converting enzyme 2 (ACE2), followed by cleavage by the enzyme transmembrane serine protease 2 (TMPRSS2), which allows the virus to enter the host cell. The ACE2 receptor and TMPRSS2 have been found in epithelial cells of the salivary gland mucosa [2,3] and the entire oral cavity [4]. Oral cavities are one of the first gateways of virus entry into the body. Thus, their role appears to be crucial for the onset and progression of infection [3]. Some researchers have also described possible links between acute respiratory infection and inflammation of the Eustachian tube, middle ear mucosa, and otitis media which can lead to hearing loss [5]. The periodontal tissue of periodontitis patients overexpresses the ACE2 receptor and the TMPRSS2 coreceptor [6].

COVID-19 is a disease known as an acute respiratory distress syndrome [7]. The disease may be asymptomatic or present with severe pneumonia leading to respiratory failure [8]. COVID-19 also manifests itself in the mouth; the symptoms include blisters, vesicles, erosions, ulcers, plaque, halitosis, verrucous tongue, pustules, petechiae, and even necrosis. Spontaneous bleeding may also occur [9]. The risk of severe disease and death increases with age and with the presence of diseases such as obesity, diabetes, and hypertension. Most of the common risk factors for severe sequelae of COVID-19 are strongly associated with poor oral health, tooth loss, and periodontitis; this fact indicates a link between oral health and general health in COVID-19 patients and the severity of this disease [10,11]. Studies were therefore launched to confirm this hypothesis. However, in a case–control study, periodontitis alone was shown to be a prognostic determinant of severe COVID-19 survival, and its co-existence was associated with hospitalization in the intensive care department, the use of mechanical ventilation, and mortality [6,12]. The same results were found in a study that included not only periodontal status but also other oral health criteria, such as caries incidence and prosthetic use [10]. However, in a meta-analysis of epidemiological studies, the higher tendency for SARS-CoV-2 infection among people suffering with periodontitis compared to those with a healthy periodontium was not statistically confirmed [6].

Due to the rapid spread of the virus, there was a need to develop effective strategies to reduce the risk of disease among the population. Immunization is considered the best form of protection against infectious diseases [13]. Three doses of the SARS-CoV-2 vaccine and a booster jab were available in Poland. Polish citizens had the opportunity to be vaccinated with Pfizer, Moderna, AstraZeneca, and Johnson & Johnson vaccines [14].

Significant contributions in the area of patient and personnel protection can be attributed to the increasing availability, especially toward the end of the pandemic, of rapid diagnostic tests that made it possible to assess in a simple, screening manner the virological status of patients admitted for emergency dental care [15].

Caries is the most widespread disease of the masticatory system. It is an infectious disease of the hard tissues of teeth with a complex etiopathomechanism. In preventing caries, a role is played not only by professional prophylaxis, but also, and, in fact, above all, by proper daily dental hygiene at home and maintaining a reasonable dietary regime.

The WHO includes oral health-related quality of life as part of the Global Oral Health Program. The condition of an oral cavity affects the functioning of the entire body. Patients who regularly visit the dentist show better a quality of life, have less pain connected with oral cavities such as teeth, oral mucosa, gums, tongue, and temporomandibular joint [16]. Patients with poor oral health and inadequate oral hygiene are susceptible to and predicted to have lower self-esteem, life satisfaction and self-acceptance, as well as poor general health and take worse care of themselves and their health [17].

According to M. Lalonde, the environment in which the patient lives is also responsible for 21% of their state of health [18]. With this in mind, it was decided to include the place of residence and the distance between the place of residence and the dental institute in this study. 

Our study aims to investigate the correlation between certain socio-economic factors, oral health care, and the receipt of COVID-19 vaccine doses. Our study analyzed the dental status of patients presenting for treatment during the coronavirus pandemic and analyzed the association with vaccination against COVID-19. The relationship between oral cavities and the SARS-CoV-2 virus is an issue that has been widely analyzed and studied. This study aims to investigate this aspect of patients’ oral health attitudes and attitudes towards COVID-19 vaccination during the pandemic.

## 2. Materials and Methods

An opinion of the Bioethics Committee on the non-requirement of approval for retrospective studies was obtained (RNN/209/23/KE). The presence of caries and its clinical manifestation as well as the number of retained teeth was used as a criterion for assessing the oral health of patients.

In this study, 2034 medical histories of patients treated in the Dental Institute of the Medical University of Lodz during the SARS-CoV pandemic between 2020 and 2022 were analyzed. We considered the number of total teeth, the number of filled teeth, the number of decayed teeth and the number of extracted teeth excluding third molars. The absence of third molars in clinical examination may have different causes and should not be equated with the absence of other teeth in the dental arch.

For each patient, DMFT and dental treatment index indicators were analyzed. DMFT is a highly valuable index for assessing and monitoring oral health status, where D stands for the number of decayed teeth, M, number of teeth extracted due to caries, and F, teeth with dental fillings.

We also analyzed the intensity of treatment individually. The restorative is the number of filled teeth divided by the total number of filled teeth and those with caries, expressed in percentages. In addition, number of doses of COVID-19 vaccine with which the patient was vaccinated and the dates of their administration since the introduction of the vaccine in Poland were analyzed. Patients’ residence was analyzed (large city > 100,000 residents, medium city 20,000–100,000 residents, small city < 20,000 residents, rural area) as well as the distance from their residence to the institute of Dentistry. Statistical analysis was performed in Statgraphics Centurion 18 (Statgraphics Technologies Inc., The Plains, VA, USA). A *p*-value < 0.05 was taken as statistically significant.

## 3. Results

**Figure 1:**
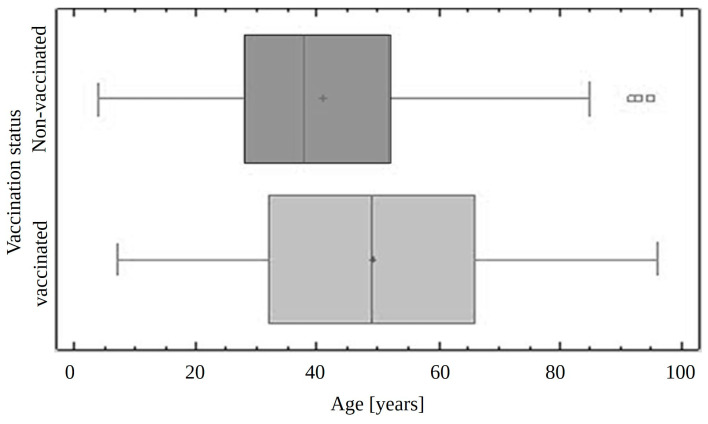
Median age in two groups of patients: non-vaccinated and vaccinated.

Among the people surveyed, 655 of them were unvaccinated while 1368 were vaccinated with at least one dose of the vaccine. This results in a vaccination rate of more than 67%. In the group of vaccinated patients, the number of vaccination doses received ranged between 1 and 4, with an average of 1.74 ± 1.39, and a median of 2. The ages of the patients studied ranged from 4 to 96 years and it was similar in both groups (4 to 95 years old in non-vaccinated and 7 to 96 years in vaccinated). In the unvaccinated group, the median age was 38, while in the vaccinated group it was 49 (*p*-value < 0.05) (Figure 1). DMFT and Dental Treatment indexes were measured for each group, excluding patients with no permanent teeth (patients < 7 years old).

**Figure 2:**
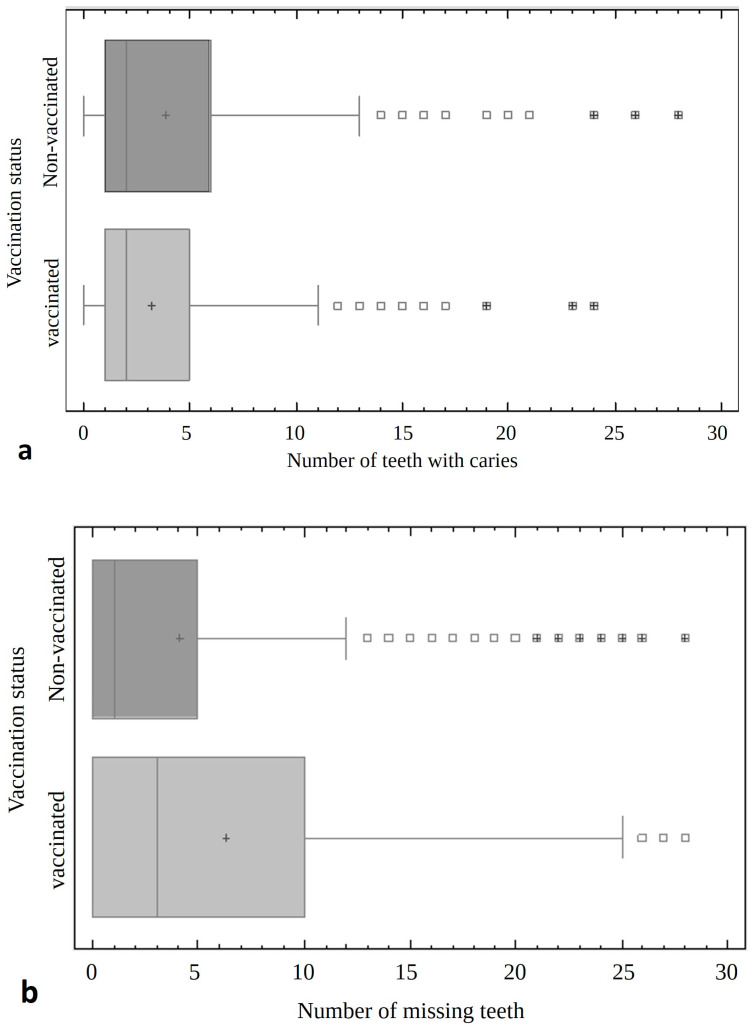

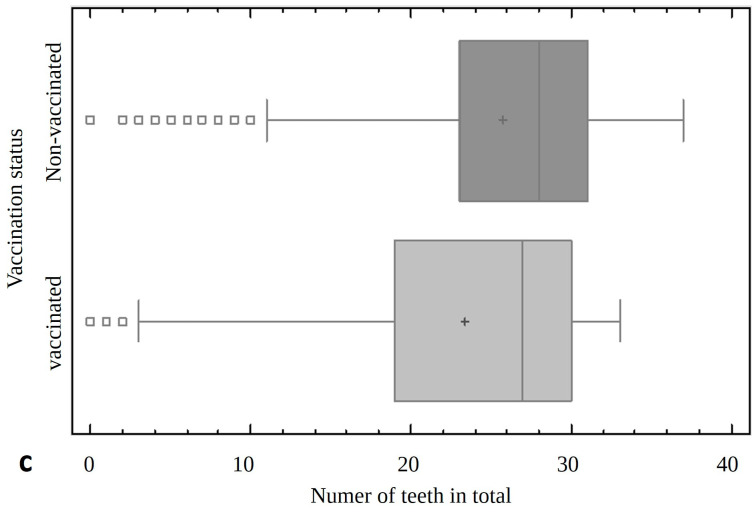
The comparison of non-vaccinated and vaccinated patients with regard to the of number of teeth with caries (**a**), number of missing or extracted teeth (not including wisdom teeth) (**b**), and number of total teeth (**c**).

Statistically, the unvaccinated patients had significantly more teeth with caries (mean 3.88 ± 4.33) and less teeth extracted (4.12 ± 6.21) compared to vaccinated patients (mean 3.19 ± 3.53, 6.3 ± 7.61) (*p* < 0.001). They also had a higher number of total teeth (25.74 ± 7.18) compared to vaccinated patients (23.36 ± 8.57) (*p* < 0.00001) (Figure 2).

**Figure 3:**
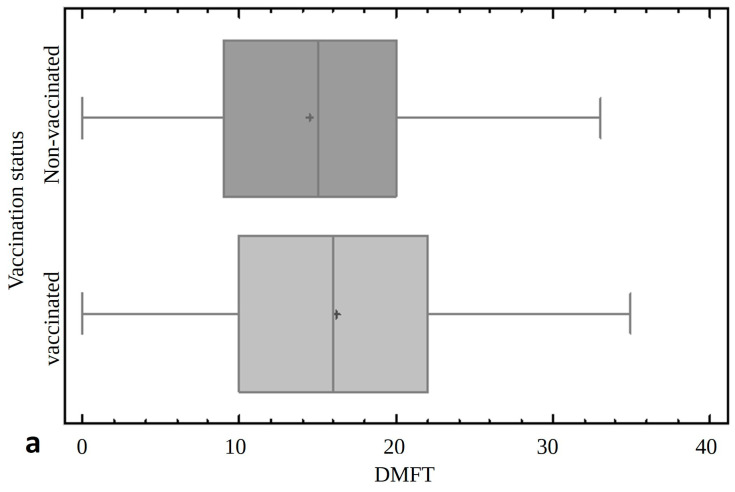

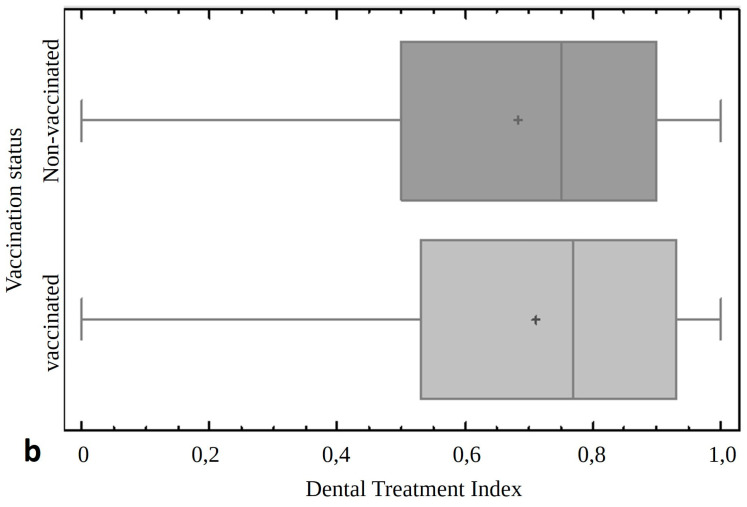
The comparison of non-vaccinated patients and vaccinated patients with regard to DMFT indicator (**a**) and Dental Treatment Index (**b**). DMFT indicator: D—number of decayed teeth; M—number of extracted teeth due to caries; and F—teeth with dental fillings.

Statistically, vaccinated patients had a significantly higher DMFT index (16.18 ± 7.78) compared to vaccinated patients (14.49 ± 7.68) (*p* < 0.0001). They also had a statistically and significantly higher Dental Treatment Index (68%) compared to vaccinated patients (71%) (*p* < 0.05) (Figure 3).

The total number of teeth and number of teeth with caries decreased with age (*p* < 0.0001). The correlations are described by the following formulas:

$$Number\;of\;teeth=\sqrt{{\left(p-q\times Age[years]\right)}^{2}}\;,\;Decayed\;teeth={\left(p-q\times Age\left[years\right]\right)}^{2}$$

**Figure 4:**
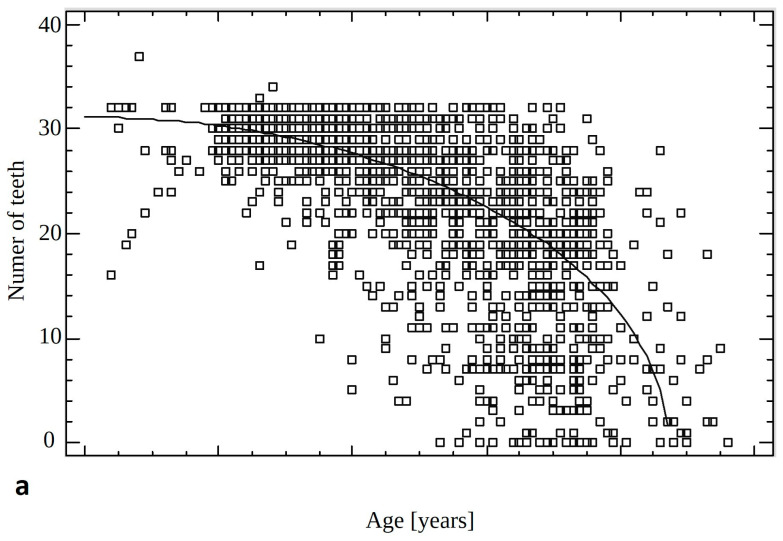

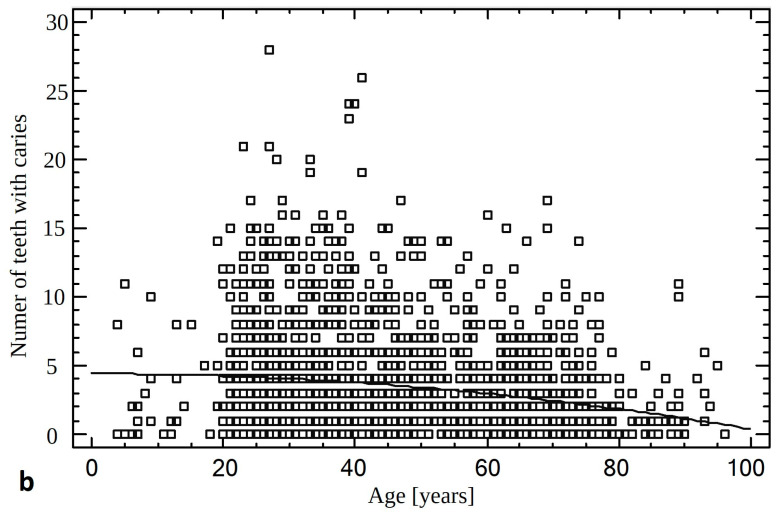
Results for the number of teeth in total (**a**) and number of teeth with caries (**b**) in the correlation with age of patients.

The *p* and *q* coefficients introduced to simplify the formulas are, for Number of teeth, *p* = 969.548 and *q* = 0.127726; for Decayed teeth *p* = 4.42163 and *q* = 0.000402749, correspondingly (Figure 4).

It has been observed that with the number of extracted and filled teeth, extracted teeth as well as the DMFT indicator increases with age (*p* < 0.0001). The correlations are described by the following formulas:

$$Teeth\;with\;dental\;fillings=p-\frac{q}{Age[years]}\;Extracted\;teeth={\left({p+q\times Age[years]}^{2}\right)}^{2}\;DMFT=p+q\sqrt{Age[years]}$$

**Figure 5:**
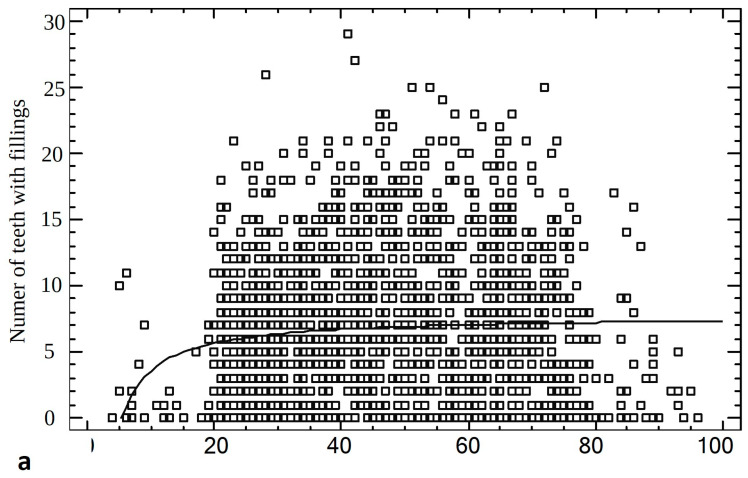

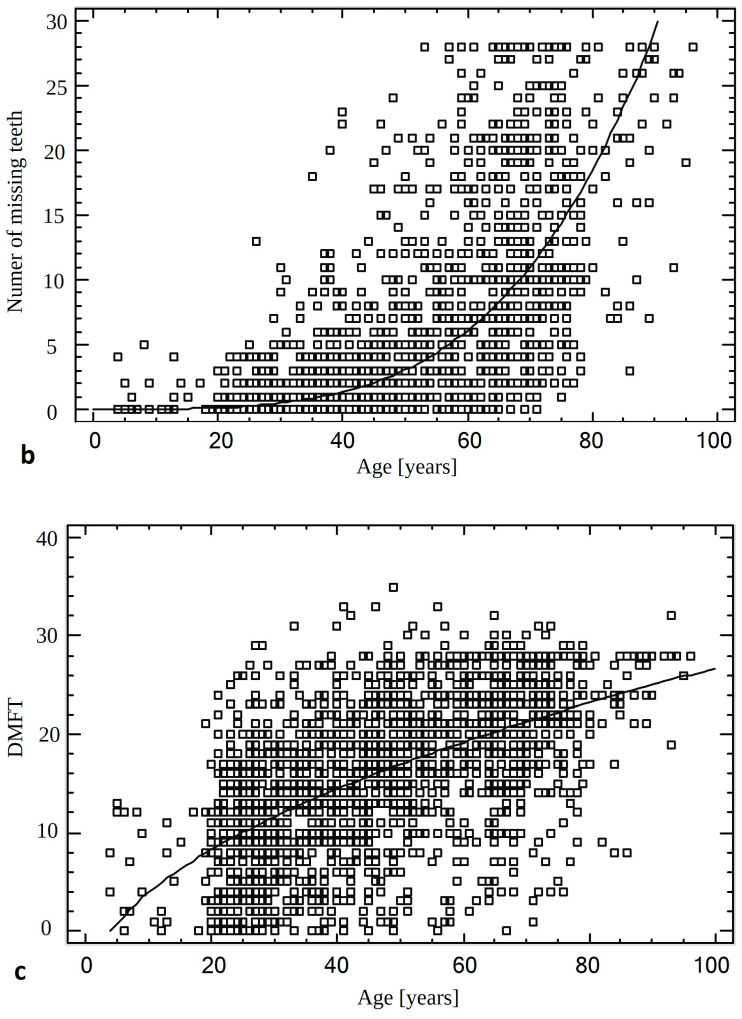
Results for the number of teeth with dental fillings (**a**), number of missing teeth/extracted teeth (not including wisdom teeth) (**b**), and the DMFT indicator (**c**) in correlation with age of patients. DMFT indicator: D—number of decayed teeth; M—number of teeth extracted due to caries; F—teeth with dental fillings.

The *p* and *q* coefficients introduced to simplify the formulas are, for Teeth with dental fillings, *p* = 7.75304 and *q* = 42.6675; for Extracted teeth they are *p* = 0.121161 and *q* = 0.00065227; and for DMPF they are *p* = 6.65446 and *q* = 3.33519, correspondingly (Figure 5).

**Figure 6:**
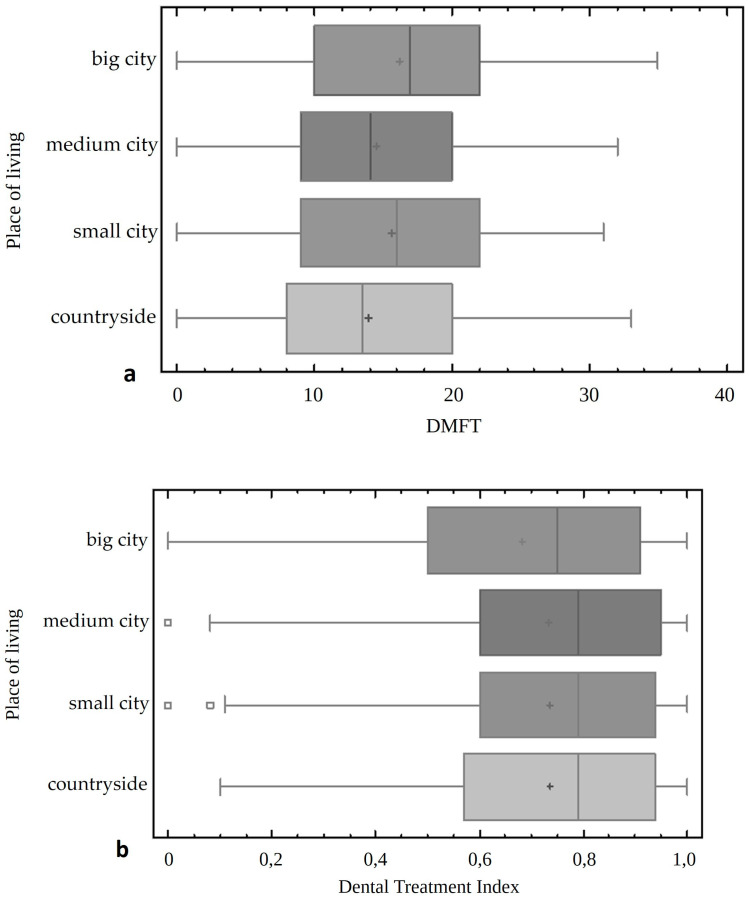
The comparison of the DMFT indicator (**a**) and the Dental Treatment Index (**b**) in people from different places of living.

Our study showed a lower DMFT index and higher restorative index in people from the countryside compared to those living in a large city (*p* < 0.0001), whereby the Dental Treatment Index was statistically significantly lower (Figure 6).

## 4. Discussion

In Poland, the largest age group vaccinated against COVID-19 is those aged 61–70 (11 million people) [14]. There are more than 22,870,000 people nationwide who have been vaccinated with at least one dose [14], which is less than 61% of the population. Thus, the vaccination rate among the Institute of Dentistry patients was slightly higher than in the overall Polish population. In our study, among those vaccinated, the median age was 49 years. When compared to nationwide vaccinations, such an age group ranks second in number, with more than 9 million of those vaccinated being 41–50 years old [14].

Statistically, the number of teeth with decayed, filled, and extracted teeth and the DMFT index increase significantly with the age of the patients. Noteworthy is the manner of decline in the number of teeth, in general, and teeth with caries. The highest number of teeth with caries was observed in adolescent patients (<20 years old). Above the age of 20, the number of teeth with caries decreased, which is probably related to the decreasing total number of teeth.

With age, an increase in the number of filled teeth was noted until the age of 30, after which it remained constant. At the same time, a steady exponential increase in the number of lost teeth was observed above the age of 30 up to which it remained relatively constant. DMFT, on the other hand, showed a logarithmic increase. The first increase in the DMFT index occurs during adolescence, while it increases much later in adults [19], which has been proven in many studies. Our study also confirmed this thesis. One can see a significant increase in the DMFT index among the elderly, which increases with age. Due to the development of caries in the crowns of teeth and roots, there is a need for fillings or the extraction of teeth due to caries.

Our study shows that unvaccinated patients have fewer teeth with caries and fewer teeth extracted due to caries than vaccinated patients. The statistically significant differences between the two groups are most likely due to the age difference between the patients in the two groups: the vaccinated were older than the unvaccinated.

On the basis of our study, vaccinated people have more filled teeth than unvaccinated people, which is a logical explanation for the fact that the rate of treatment intensity among vaccinated people is higher than in unvaccinated people. Further evidence confirming the hypothesis of better oral health care among vaccinated patients was found in the statistically and significantly higher rate of conservative treatment among vaccinated patients. However, a direct relationship between vaccination and oral health has not yet been proven. The statistically significant differences between the two groups are most likely due to the age difference.

The relationship between the DMFT index and intensity of treatment and area of residence is interesting. In 2017, in an epidemiological study of the Polish adult population aged 35–44 in a group of randomly surveyed patients, a positive correlation between the number of preserved natural teeth was observed with the co-occurrence of such factors as living in urban areas, higher education, and more favorable material situation. Our study showed a lower average DMFT index and a higher treatment intensity index in people from the countryside than in those living in a large city. In this regard, our study stands in opposition to the existing research findings [20]. The likely reason for the differences in these results is that our study was conducted during the pandemic and lockdown. From rural areas, motivated patients during the course of treatment already started before the pandemic reached the institute, while from the urban area mainly patients with pain arrived.

The problem of patients’ motivation to undertake treatment, who chose to undertake treatment at a more advanced stage of the disease, is also shown in a study by Grill et al. [21]. The author presented results showing an increased number of patients with very advanced abscesses requiring treatment under general anesthesia compared to abscesses incised under local anesthesia than before the pandemic. Our study also noted an increased number in patients presenting with an acute condition (toothache) especially in an urban area. Other factors affecting oral health are known, of course. Factors such as low socio-economic status and unhealthy lifestyles (smoking and alcohol consumption) are known factors associated with poorer oral health [18,19]. The authors did not have access to the above data.

This was also reflected in the oral health of patients from urban areas. In our study, statistically, patients living in a large city had significantly fewer teeth than patients from small towns and rural areas.

Population studies conducted in the same area in non-pandemic times show that such factors as urban residents, higher education, declared above-average financial situation, gender, and sex were correlated with higher number of teeth. To make a definite statement, further research is needed on the connection between oral health and general health expressed, manifested by undergoing immunizations against infectious diseases of individuals. The role of dentists should be prominent in raising awareness of how untreated caries or periodontitis affect the course of diseases, including COVID-19, as well as motivating their patients to take preventive measures such as immunizations, which are essential to maintain a good health condition. Most of the available studies have not found a correlation between patients’ place of residence, but Garcés-Elías noted in his study that rural residents are more likely to have health insurance, compared to those living in urban areas. On the other hand, he highlighted better hygiene habits such as tooth brushing in urban residents compared to rural areas [22].

This topic requires further analysis based on multicenter cohort studies of the time of the COVID-19 pandemic.

## 5. Conclusions

COVID-19 disease is inseparable from oral cavities. It provides a gateway for infection for the SARS-CoV-2 virus into the system. There are reports of the occurrence of periodontal disease, dental caries and poor oral hygiene as a risk factor for the disease and a more severe course of infection. Furthermore, the disease also has its clinical manifestation in oral cavities. Dental prophylaxis and possible effective treatment, along with other healthy lifestyle principles, can perform an important role in the prevention of this infectious disease.

Our study did not show the presence of a definite correlation between high vaccination rates against COVID-19 and good oral health, nor between low vaccination rates and poor oral health. These associations may be influenced by other factors discussed above. Further research on this topic would be needed.

It is the dentist that patients see more often than other doctors, so it is incumbent on them to educate not only about keeping satisfactory oral hygiene, but also about other health-promoting behaviors such as receiving vaccinations.

## Data Availability

The original data presented in the study are openly available in https://doi.org/10.6084/m9.figshare.25805353.v1.
